# Juvenile rhupus syndrome: A case report of coexistence of JIA and SLE

**DOI:** 10.1097/MD.0000000000047279

**Published:** 2026-01-23

**Authors:** Zain Ul Abedeen, Zia Ullah, Wajeeh Ur Rehman, Naeem Ullah, Amal Elalfy, Kamil Ahmad Kamil, Muhammad Kashif Habib

**Affiliations:** a Department of Internal Medicine, Saidu Medical College, Swat, Pakistan; b The British University in Egypt, El Shorouk, Egypt; c Department of Internal Medicine, Mirwais Regional Hospital, Kandahar, Afghanistan.

**Keywords:** autoimmune diseases, case report, juvenile idiopathic arthritis, overlap syndromes, rhupus syndrome, systemic lupus erythematosus

## Abstract

**Background:**

Juvenile rhupus syndrome is the coexistence of juvenile idiopathic arthritis (JIA) and systemic lupus erythematosus (SLE) in a pediatric patient. It is a rare but clinically significant autoimmune overlap syndrome.

**Case:**

We present a case of a 10-year-old girl (initially diagnosed with rheumatoid factor-positive polyarticular JIA and being treated with methotrexate) who developed features suggestive of an SLE flare-up and/or methotrexate toxicity, including pallor, non-scarring alopecia, dyspnea, pancytopenia, impaired liver function tests, and restricted mobility in the elbow and knee joints; however, laboratory reports indicated elevated antinuclear antibody and anti-double-stranded DNA antibodies titers, as well as decreased complement levels. After thorough clinical assessment and excluding other potential causes, she fulfilled the 2019 European League Against Rheumatism /American College of Rheumatology criteria for SLE diagnosis, leading to the diagnosis of juvenile rhupus syndrome. Such disease evolution required therapeutic reassessment, and her immunosuppressive regimen was revised.

**Conclusion:**

Rhupus syndrome is just as significant in children as it is in adults, where it is more commonly reported. Early recognition and adequate therapy are essential for improved clinical outcomes as juvenile rhupus often shows severe systemic involvement and increased morbidity compared to isolated JIA or SLE. Our case contributes to the limited literature on juvenile rhupus and highlights the need for clear diagnostic criteria and management guidelines.

## 1. Introduction

Rhupus syndrome (a rare autoimmune overlap syndrome) was 1st reported in 1960; however, Peter Schur established the term in 1971.^[1]^ It is the coexistence of juvenile idiopathic arthritis (JIA) (or rheumatoid arthritis (RA) in case of adults) and systemic lupus erythematosus (SLE) in a patient. Either condition may develop 1st, or both may present simultaneously; however, it usually begins with manifestations of JIA, and SLE features appear later in the disease course.^[2]^

Immune responses (normally protective) help us fight against potentially harmful antigens; however, they can lead to autoimmune conditions when dysregulated, as in case of SLE. SLE is a chronic systemic autoimmune disorder characterized by the production of antibodies against self-antigens in the skin, joints, kidneys, brain, and blood. It results in tissue inflammation and degeneration affecting multiple organ systems in the body.^[3]^ The European League Against Rheumatism (EULAR)/American College of Rheumatology (ACR) criteria require a cumulative score of 10 or more in the presence of a positive antinuclear antibody at a titer of at least 1:80 for SLE diagnosis. The criteria include 7 clinical and 3 immunological domains.^[4]^

Similarly, JIA is a heterogeneous group of immune-mediated disorders. The International League of Associations for Rheumatology (ILAR) defines JIA patients as children aged <16 years, having arthritis affecting 1 or more joints for 6 weeks or more. Diagnosis relies on distinct clinical characteristics and laboratory findings in the absence of any potential cause.^[5]^ These patients suffer from arthralgia, restricted joint mobility, growth retardation, and inability to engage in physical activity, thus having lower health-related quality of life.^[6]^

Although rhupus syndrome is usually reported in adults, it is of equal significance in children. Although considered as JIA–SLE overlap, juvenile rhupus remains poorly defined and underreported, with scarce clinical data available in the current literature. Thus, our case report adds to the limited clinical evidence on juvenile rhupus and underscores the need for long-term follow-up in rheumatologic conditions. Rhupus syndrome has a distinctive clinical course, warranting timely diagnosis, prompt therapy, and further research.

## 2. Case presentation

We report a case of a 10-year-old girl who was 1st presented to us in March 2024, with complaints of fever and multiple joint pain with associated decreased range of motion, for the past 3 months. On presentation, the joints primarily involved were the bilateral metacarpophalangeal, elbow, shoulder, knee, ankle, and metatarsophalangeal joints. Initial investigations revealed erosive arthritis, elevated ESR (85 mm in the 1st hour), anemia (Hb: 10.2 g/dL; reference: 11–16 g/dL), positive rheumatoid factor (RF), and anti-cyclic citrullinated peptide antibodies (anti-CCP) Antibodies (Table 1). Her family history was positive for her father, who was diagnosed with JIA at age 10 and had been treated for over a year. All these clinical features and laboratory findings led us to diagnose polyarticular JIA, as per ILAR criteria.^[5]^ The patient was initiated on methotrexate (MTX) 10 mg/week with folic acid 5 mg/week and prednisolone 0.5 mg/kg/day in divided doses.

**Table 1:** Immune serology profile and laboratory investigations of the patient.

Immune serology		Laboratory investigations		
Test name	Results	Test name	Results	Reference range
ANA by IFA ANA titer IFA pattern	Positive 1:1280 Homogenous	ESR	23 mm/h	<15 mm/h
		ALT	100 U/L	Up to 40 U/L
Anti-nucleosome	Positive	C3	28 mg/dL	80–170 mg/dL
Anti-dsDNA	Positive	C4	4 mg/dL	12–42 mg/dL
Anti-histone	Positive	Serum creatinine	0.45 mg/dL	0.55–1.3 mg/dL
Anti-smith	Negative	eGFR	260 mL/min/1.73 m^2^	>60: normal 15–60: borderline <15: kidney failure
Anti-ribosome	Negative			
RA factor	Positive			
Anti-CCP	Positive			
Anti-RNP 68 kD/A/C	Negative			
Anti-PCNA	Negative			
Anti-SSA/Ro 60 kD	Negative			
Anti-SSA/Ro 52 kD	Negative			
Anti-SSB/La	Negative			
Anti-Scl-70	Negative			
CENP-A/B	Negative			
Anti-Pm-Scl	Negative			
Anti-MI-2	Negative			
Anti-Jo-1	Negative			
Anti-Sm/RNP	Negative			

ALT = alanine transaminase, ANA = antinuclear antibody, anti-CCP = anti-cyclic citrullinated peptide antibodies, anti-dsDNA = anti-double-stranded DNA antibodies, anti-Jo-1 = anti-histidyl-tRNA synthetase antibodies, anti-MI-2 = anti-Mi-2 antibodies, anti-PCNA = anti-proliferating cell nuclear antigen antibodies, anti-Pm-Scl = anti-polymyositis-scleroderma antibodies, anti-RNP 68 kD/A/C = anti-ribonucleoprotein antibodies (68 kD/A/C subunits), anti-Scl-70 = anti-topoisomerase I antibodies, anti-Sm/RNP = anti-Smith/ribonucleoprotein antibodies, anti-SSA/Ro 52 kD = anti-Sjögren syndrome antigen A (Ro) 52 kD antibodies, anti-SSA/Ro 60 kD = anti-Sjögren syndrome antigen A (Ro) 60 kD antibodies, anti-SSB/La = anti-Sjögren syndrome antigen B (La) antibodies, C3 and C4 = complement protein 3 and 4, CENP-A/B = centromere protein A/B antibodies, eGFR = estimated glomerular filtration rate, ESR = erythrocyte sedimentation rate, IFA = indirect immunofluorescence assay, RA = rheumatoid arthritis.

During follow-up visits over the subsequent months, the patient demonstrated gradual improvements and no known medication-associated side effects. However, she experienced decreased appetite, weight loss, and signs of growth failure. Laboratory evaluations were conducted over time to assess hematological status and hepatic and renal functions, which showed persistently elevated ESR and mild leukopenia.

**Figure 1.**
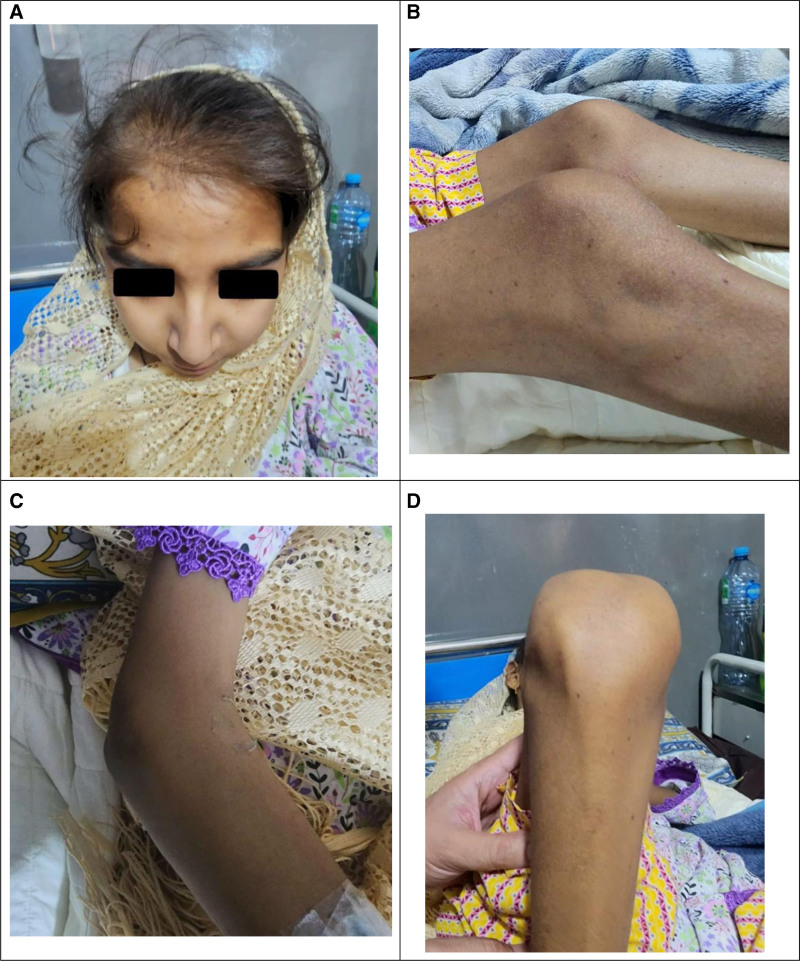
Images of the patient showing various clinical features. (A) Alopecia affecting the scalp. (B) and (C) Lateral view of the partially flexed knee and elbow joints, highlighting decreased range of motion. (D) Anterior view of the right knee showing prominent joint swelling.

**Figure 2.**
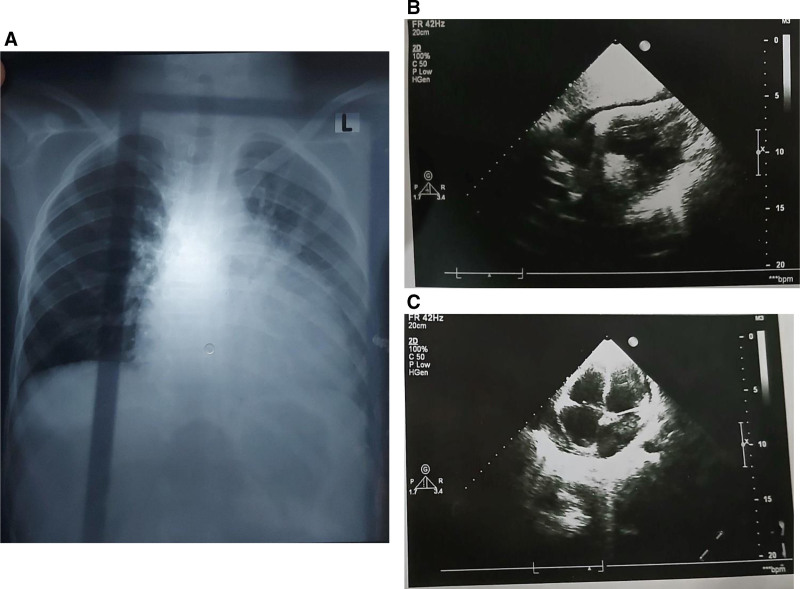
Radiographic evaluations of the patient for serositis. (A) Chest radiograph demonstrating an enlarged cardiac silhouette with clear lung fields and no pleural effusion. (B) and (C) Parasternal long- and short-axis transthoracic echocardiographic views, respectively, showing balanced cardiac chambers and a thin, circumferential pericardial effusion, with preserved systolic function.

In the recent follow-up, she exhibited pallor, non-scarring alopecia, dyspnea, and restricted mobility in the elbow and knee joints (Fig. 1). There were no signs of fever, oral or nasopharyngeal ulcers, malar butterfly rash, discoid rash, lymphadenopathy, hepatomegaly, splenomegaly, or joint tenderness. Her weight and body surface area were 26 kg and 0.96 m^2^, respectively. Routine investigations revealed pancytopenia and mildly raised alanine aminotransferase (100 U/L; normal up to 40 U/L). Relevant differentials of pancytopenia, including extensive systemic involvement in JIA, SLE flare-up, MTX-induced bone marrow suppression, aplastic anemia, acute viral infections (including Epstein–Barr virus, cytomegalovirus, or parvovirus B19), macrophage activation syndrome, and hemophagocytic lymphohistiocytosis (HLH), were considered. However, the absence of persistent high fever, hepatosplenomegaly, lymphadenopathy, and coagulopathy at presentation made the viral etiology and macrophage activation syndrome/HLH less likely. Therefore, viral serology and bone marrow examination were not obtained at this stage. Subsequent investigations were primarily directed towards SLE, due to its significant association with JIA and RA as per the established literature, and MTX toxicity due to its prolonged use.^[7]^ Her immune serology reports were positive for antinuclear antibody (homogenous pattern at a titer of 1:1280, reference: <1:180), anti-Smith antibodies, anti-double-stranded DNA antibodies, anti-nucleosome, and anti-histone antibodies (Table 1). Chest X-ray and transthoracic echocardiogram were advised for underlying dyspnea, which showed a thin rim of pericardial effusion with balanced chambers and normal ejection fraction (65%) (Fig. 2). Renal function tests were unremarkable. Reports also showed decreased complement proteins, including complement component 3 (28 mg/dL, reference: 80–178 mg/dL) as well as complement component 4 (4 mg/dL, reference: 12–42 mg/dL), and a negative direct Coombs test (Table 1). The presence of SLE-specific antibodies, decreased complement levels, along with a negative coombs test favored SLE flare over MTX toxicity. Moreover, mildly elevated alanine aminotransferase, normal renal function tests, and absence of previous evidence of hepatoxicity and myelosuppression made the MTX toxicity less likely. As the patient was on low-dose MTX weekly regimen, drug levels were not obtained. Accordingly, the patient fulfilled the revised 2019 EULAR/ACR criteria for SLE.^[4]^ This coexistence of JIA and juvenile-onset SLE supported a diagnosis of juvenile rhupus syndrome.^[7]^ Recent evaluations and current clinical status required new therapeutic decisions. The patient was hospitalized and received leucovorin rescue therapy empirically till the exclusion of suspected MTX toxicity. Upon confirmation of SLE flare, she received hydroxychloroquine (HCQ) 200 mg/day and intravenous dexamethasone 4 mg twice daily. After 4 days of monitoring and clinical improvement, she was discharged on the following outpatient regimen: hydroxychloroquine 200 mg/day, azathioprine 50 mg/day, as well as MTX 10 mg/week with 5 mg folic acid weekly. The overall clinical picture of SLE and improvement on standard SLE therapy further supported the exclusion of other differentials.

She was called for follow-up 15 days later, in which she demonstrated symptomatic improvement (including reduced joint pain and improved mobility) and thus, the same treatment plan was continued.

## 3. Discussion

Juvenile rhupus syndrome is characterized by the coexistence of JIA and SLE. Although rare, it is a clinically significant pediatric autoimmune overlap syndrome, with the patient exhibiting symptoms and laboratory findings of both conditions at the same time. Patients (usually females) present initially with features of JIA like persistent polyarthralgia, joint swelling, and stiffness with raised inflammatory markers, and develop SLE later in the clinical course, in the majority of cases.^[2,7]^ The interval between appearance of JIA and subsequent SLE ranges from several months to 4 years on average.^[2,8]^ Similarly, our case was diagnosed as RF-positive, polyarticular JIA initially, and developed SLE features (non-scarring alopecia, pancytopenia, serositis, decreased complement levels, and positive SLE serology) a year later. The clinical progression aligns with systematic reviews and multicenter studies, which report a similar clinical course.^[2,7,8]^ However, cases with initial SLE presentation or simultaneous manifestation of both conditions have also been documented.^[9]^ This discrepancy in presentation complicates diagnosis and delay in prompt treatment. It also emphasizes the importance of long-term monitoring in JIA patients.

Comparative studies also show that rhupus patients frequently have more severe articular, cutaneous (66.7–80%), and systemic involvement than those with isolated SLE or JIA, with higher rates of hematological (53.4–58.3%), renal (23.3–64%), and serosal complications.^[2,10,11]^ Arthritis is common among all rhupus patients, which is typically symmetric, erosive polyarthritis indistinguishable from RA/JIA. In contrast, SLE with excessive articular involvement is characterized by non-deforming, non-erosive arthritis and lacks RA/JIA serologic findings. This distinguishes rhupus syndrome from SLE-related arthritis.^[7,8]^ Our patient exhibited excessive articular features along with hematologic and pericardial involvement. The coexistence of JIA and SLE features and the temporal sequence of clinical progression in our patient supported the diagnosis of juvenile rhupus, instead of SLE-related arthritis or JIA with excessive systemic involvement.

Due to extensive clinical features and concurrent complications, management of pediatric rhupus is often challenging and requires an individualized approach tailored to disease severity and organ involvement. In the majority of reported cases, a combination of immunosuppressants been utilized.^[2,7,8]^ Upon diagnosis of rhupus, our patient received dexamethasone and HCQ as an inpatient regimen and a combination of MTX, HCQ, and azathioprine as an outpatient regimen. The intravenous dexamethasone is a preferred agent to control SLE flare-up, significant serositis, and hematologic complications, while other immunemodulators work more slowly.^[12]^ As per the EULAR guidelines, HCQ should be considered in all SLE patients, unless contraindicated, as it reduces disease flare, prevents organ damage, and improves long-term survival.^[13]^ Also, Azathioprine was initiated as it controls extra-articular manifestations of the disease and provides a steroid-sparing effect.^[13]^ After excluding the MTX toxicity, we continued MTX as an outpatient regimen as it has been shown to demonstrate efficacy in managing the articular and mucocutaneous manifestations of both JIA and SLE. A systematic review and meta-analysis report that MTX use in SLE significantly reduces the disease activity index and corticosteroid requirement.^[14]^ Following the current regimen, our patient showed signs of improvement on her 1st follow-up, after being diagnosed with rhupus.

Although the prognosis of juvenile rhupus remains unclear due to its rare occurrence and scarce long-term data, the extent and severity of disease, early diagnosis, and suitable treatment are important factors affecting long-term outcomes. Studies suggest increased morbidity compared to SLE or JIA, especially when associated with severe organ involvement.^[2,7,8]^ For instance, a case series by Ziaee et al reported that one of the patients died despite aggressive therapy, due to extensive systemic involvement.^[7]^ Timely recognition and appropriate therapeutic approach are essential for better clinical outcomes.

Rhupus syndrome has been reported primarily in adults. It is equally significant in the pediatric population, but studies directly comparing the two are sparse. However, a recent study by Sener et al offered a direct comparison between the two and reported significant differences. Juvenile rhupus is reported to have a shorter interval between JIA and SLE diagnosis and a higher rate of cutaneous, renal and neuropsychiatric involvement compared to adult rhupus syndrome. anti-double-stranded DNA antibodies positivity is more common in pediatrics, while anti-CCP is more frequent in adults patients. In pediatrics, drugs like MTX, corticosteroids, and hydroxychloroquine have been effectively used, while rituximab and leflunomide are preferred in adults.^[11]^ Upon diagnosis of rhupus syndrome, our patient’s immunosuppressant regimen was also revised as per the established literature.

Our case report also has some limitations due to the low-resource settings and low socioeconomic status of the patient. Serum MTX levels were not obtained as therapeutic drug monitoring is not routinely performed for low-dose weekly MTX regimens. Additionally, bone marrow examination was also not obtained, as the clinical profile favored SLE flare-up. Also, the radiographic images documenting the erosive arthritis observed at initial presentation are unavailable; however, high anti-CCP antibody titers and presence of RF positivity strongly support the erosive nature of the arthritis, as anti-CCP antibodies have been strongly associated with erosive arthritis in both JIA/RA and rhupus patients.^[15]^

## 4. Conclusion

In summary, this case contributes to the limited literature on JIA and SLE overlap (termed as juvenile rhupus syndrome; a clinically significant autoimmune overlap) and highlights the importance of considering overlap syndrome in children presented with unusual presentation and/or evolving features. It emphasizes the need for future research to establish diagnostic criteria and management strategies for juvenile rhupus syndrome. Rhupus, or JIA and SLE alone, are distinct conditions that require prompt diagnosis and tailored therapy for better clinical outcomes.

## Acknowledgments

We are thankful to the patient and her family for their co-operation and giving consent for participation in the case report. We also appreciate the participation of multidisciplinary team engaged in her care.

## Author contributions

**Conceptualization:** Zain Ul Abedeen, Zia Ullah, Wajeeh Ur Rehman, Naeem Ullah.

**Data curation:** Zia Ullah, Naeem Ullah, Amal Elalfy, Kamil Ahmad Kamil, Muhammad Kashif Habib.

**Investigation:** Zain Ul Abedeen, Wajeeh Ur Rehman, Naeem Ullah, Muhammad Kashif Habib.

**Methodology:** Zain Ul Abedeen, Zia Ullah, Kamil Ahmad Kamil.

**Project administration:** Zain Ul Abedeen, Wajeeh Ur Rehman, Naeem Ullah.

**Supervision:** Wajeeh Ur Rehman, Naeem Ullah.

**Validation:** Wajeeh Ur Rehman, Naeem Ullah.

**Visualization:** Zain Ul Abedeen, Zia Ullah, Amal Elalfy.

**Writing – original draft:** Zain Ul Abedeen, Zia Ullah, Amal Elalfy, Kamil Ahmad Kamil.

**Writing – review & editing:** Zain Ul Abedeen, Zia Ullah, Wajeeh Ur Rehman, Naeem Ullah, Kamil Ahmad Kamil, Muhammad Kashif Habib.
